# ECG Evolution in Elite Gymnasts: A Retrospective Analysis of Training Adaptations, Risk Prediction, and PPE Optimization

**DOI:** 10.3390/diagnostics15081007

**Published:** 2025-04-15

**Authors:** Alina Maria Smaranda, Adela Caramoci, Teodora Simina Drăgoiu, Ioana Anca Bădărău

**Affiliations:** 1 Discipline of Sports Medicine, Carol Davila University of Medicine and Pharmacy, 050474 Bucharest, Romania; adela.caramoci@umfcd.ro; 2 National Institute of Sports Medicine, 022103 Bucharest, Romania; 3 Faculty of Medicine, Carol Davila University of Medicine and Pharmacy, 050474 Bucharest, Romania; teodora_simina_ionescu@yahoo.com; 4 Department of Physiology, Carol Davila University of Medicine and Pharmacy, 050474 Bucharest, Romania

**Keywords:** ECG screening, pre-participation evaluation, elite gymnasts, cardiac adaptation, predictive modeling, Monte Carlo simulation

## Abstract

**Background:** Electrocardiographic (ECG) screening is crucial in pre-participation evaluations (PPEs) for elite athletes, aiding in the early detection of cardiac adaptations and potential risks. Elite female gymnasts experience unique cardiovascular adaptations due to intensive training, yet limited longitudinal data exist on their ECG evolution. This study introduces Oracle Crystal Ball, a predictive tool for forecasting ECG abnormalities and assessing PPE cost-effectiveness to optimize screening protocols. **Methods:** This retrospective cohort study analyzed ECG and cardiovascular parameters in twelve elite female gymnasts who underwent up to 14 PPEs over several years at the National Institute of Sports Medicine, Romania. Longitudinal ECG trends, training variables, and biochemical markers were examined using statistical analyses, including logistic regression, repeated measures ANOVA, and time-series forecasting (ARIMA). Monte Carlo simulations assessed the cost-effectiveness of 6-month vs. 12-month PPE schedules. **Results:** The athletes exhibited significant cardiovascular adaptations, including progressive declines in resting heart rate and training-induced ECG changes. Junctional escape rhythms and T-wave inversions (V1–V3) increased with age, requiring refined ECG interpretation. Predictive modeling demonstrated the feasibility of individualized risk stratification, while a cost-effectiveness analysis revealed that a 12-month PPE schedule was financially advantageous without reducing diagnostic accuracy. **Conclusions:** Longitudinal ECG monitoring and predictive analytics improve risk assessment in elite gymnasts. Oracle Crystal Ball enhances athlete-specific screening, minimizing unnecessary tests while ensuring early detection of clinically significant ECG changes. A 12-month PPE schedule is a cost-effective alternative for elite athletes.

## 1. Introduction

Electrocardiographic (ECG) screening plays a critical role in pre-participation evaluations (PPEs) for elite athletes, aiding in the early detection of cardiac adaptations and the potential risks associated with intensive training [1]. Gymnasts, due to the high-intensity, early specialization, and long-term nature of their training, undergo significant adaptations, which require close monitoring [2]. Despite the widespread implementation of PPEs, there remains limited data on how ECG and cardiovascular parameters evolve over time in this population and how best to predict individual risk. While some ECG findings, such as sinus bradycardia, junctional escape rhythms, or repolarization changes, are well-documented in athletes, distinguishing benign adaptations from early pathological processes remains a major clinical challenge. Recent research highlights the need for refined ECG interpretation in pediatric athletes to distinguish physiological adaptations from potential cardiac pathology [3,4]. Furthermore, optimizing the frequency of PPEs is essential, as too frequent evaluations may lead to unnecessary testing and increased healthcare costs, while infrequent screening risks missing critical abnormalities [5,6]. This study introduces Oracle Crystal Ball as a novel predictive tool to enhance longitudinal ECG monitoring and risk stratification in elite gymnasts. Unlike conventional PPE-based screening, Oracle Crystal Ball enables individualized forecasting of future ECG abnormalities, incorporating athlete-specific training variables, biochemical markers, and autonomic regulation parameters. By leveraging advanced predictive analytics and Monte Carlo simulations, this approach could revolutionize athlete cardiac monitoring by shifting from reactive assessment to proactive prevention.

This study has three primary objectives:

○ to assess the longitudinal evolution of cardiovascular and ECG parameters in elite female gymnasts over multiple PPE cycles;
○ to develop predictive models for ECG abnormalities, utilizing Oracle Crystal Ball to forecast individualized athlete risk based on historical and physiological data;
○ to evaluate the cost-effectiveness of different PPE schedules (6 month vs. 12 month) using Monte Carlo simulations and determine the optimal screening frequency for elite athletes.

The following hypotheses were formulated to correspond to the study objectives and to guide the structure of the predictive and statistical analyses:

○ training-related cardiovascular adaptations, including declines in resting heart rate and ECG pattern shifts, will be observed over time;
○ certain training load variables and biochemical markers (e.g., creatine kinase, electrolyte balance, and VO2 max) will serve as predictors of ECG changes;
○ Oracle Crystal Ball will accurately forecast the likelihood of an athlete developing an abnormal ECG, enabling personalized risk assessment and proactive interventions.
○ a 12-month PPE schedule will be more cost-effective than a 6-month schedule, without compromising clinically relevant case identification.

By integrating longitudinal ECG tracking, predictive modeling, and cost-effectiveness analysis, this study pioneers the use of Oracle Crystal Ball for personalized athlete cardiac forecasting, offering a data-driven approach to optimizing screening protocols and preventing cardiac issues in elite athletes.

## 2. Materials and Methods

### 2.1. Study Design

This study is a retrospective cohort analysis investigating the evolution of training characteristics, cardiovascular parameters, and ECG findings in elite female gymnastics athletes over time. Data were collected from medical records at the National Institute of Sports Medicine, Bucharest, Romania, where athletes underwent pre-participation evaluations (PPEs) every six months, as required by Romanian sports legislation. The study aimed to track longitudinal changes in physiological, biochemical, and cardiovascular adaptations. The cohort was followed retrospectively from 2024 back to their first pre-participation evaluation at the National Institute of Sports Medicine, covering multiple consecutive PPE cycles between 2017 and 2024.

### 2.2. Participants

Twelve elite female gymnasts were retrospectively selected based on eligibility criteria, including:

- first PPE conducted at the National Institute of Sports Medicine (athletes had previous PPEs elsewhere, but only data from their first assessment at the Institute onward were included);
- consistent biannual PPE attendance at the institute throughout the study period;
- active competitive status at the national level during the entire follow-up period;
- no history of cardiovascular or systemic comorbidities prior to their first PPE at the institute;
- no prior exclusion from competitive sports due to medical concerns.

Athletes were excluded if they missed scheduled PPEs during the observation period, had documented comorbidities before their first PPE at the Institute, had a history of congenital heart disease, structural cardiac abnormalities, or were on cardiovascular medications

### 2.3. Variables and Outcomes

The exposure variables were the following:

- Training characteristics: training age, periodization phase, number of sessions per week, session duration, and weekly training volume;
- Biochemical markers: creatine kinase (CK), alanine aminotransferase (ALT), serum magnesium, serum calcium, total protein, urea, and glucose;
- Cardiovascular parameters: resting heart rate (HR), blood pressure at rest and after standing, heart rate recovery post-exercise, and VO_2_ max;
- Outcome variables;
- ECG findings: arrhythmias, conduction abnormalities, T-wave inversions (TWI), and incomplete right bundle branch block (IRBBB);
- Cardiac risk score: derived from cumulative ECG findings across assessment periods;
- Training adaptations: changes in HR, VO_2_ max, and autonomic regulation markers over time;
- ECG screening was performed in accordance with the 2017 International Criteria for Electrocardiographic Interpretation in Athletes [4]. Based on these criteria, ECG results were categorized as follows: 0—normal, 1—abnormal, and 2—borderline.

### 2.4. Data Collection and Measurements

- ECG analysis: standard 12-lead ECG was recorded at rest, with findings classified as normal, borderline, or abnormal based on international athlete screening criteria;
- VO_2_ max estimation: Astrand–Rhyming test used to determine aerobic capacity;
- Heart rate and blood pressure: measured at rest, during the Schellong test, at minute 6 of the Astrand test, and three minutes post-Astrand test;
- Biochemical assessments: venous blood samples were analyzed for CK, ALT, magnesium, calcium, total protein, urea, and glucose;
- Anthropometric measurements: height, weight, and body composition were assessed using the five-skinfold method;
- Training-related variables: data on training age, competition stage (competitive, pre-competitive, preparatory), weekly training volume, and the number of training sessions per week were recorded;
- Additional cardiovascular evaluations: if ECG findings warranted further assessment, echocardiography, cardiopulmonary exercise testing (CPET), 24 h Holter ECG monitoring, and electrophysiological studies were conducted to evaluate cardiac function more comprehensively.

### 2.5. Bias and Confounding Control

Selection bias was minimized by including only athletes with complete PPE records from their first assessment at the National Institute of Sports Medicine. Measurement bias was controlled through standardized assessment protocols for ECG, biochemical testing, and training logs. Confounding factors such as age, training intensity, and hydration status were adjusted through stratification and repeated-measures analysis.

### 2.6. Ethical Considerations

The study protocol was approved by the Institutional Review Board of the National Institute of Sports Medicine, Bucharest (427/10 February 2025) and conducted in accordance with the Declaration of Helsinki. Written informed consent was obtained from all participants.

### 2.7. Data Availability and Reproducibility

The dataset used for this study is available upon request.

Data collection followed strict anonymization protocols, ensuring compliance with ethical and data protection guidelines.

### 2.8. Statistical Analysis

A combination of descriptive, inferential, and predictive modeling techniques was applied:

- Pearson’s correlation and Chi-square tests: to assess associations between training variables, ECG abnormalities, and cardiovascular markers;
- Logistic regression analysis: to identify predictors of arrhythmias and conduction abnormalities;
- Repeated measures and mixed-model ANOVA: to evaluate longitudinal trends in HR, ECG classifications, and training-related adaptations;
- Time-series forecasting (ARIMA and exponential smoothing): to predict HR trends and ECG classifications;
- Monte Carlo simulation: to compare the cost-effectiveness of 6-month vs. 12-month PPE schedules;
- Break-even analysis: to determine the minimum number of detected cardiac abnormalities required to justify each PPE frequency.

All statistical analyses were performed using IBM SPSS Statistics (v29, trial license), Oracle Crystal Ball (v11.1.2.4, trial license), Python (v3.11), and Microsoft Excel (Windows 11), with significance levels set at *p* < 0.05 for hypothesis testing. Python was utilized for heatmap visualizations and advanced data modeling, while Excel was used for initial data structuring and exploratory analysis. SPSS was used for descriptive statistics, Pearson’s correlation, Chi-square tests, logistic regression, ANOVA (including repeated measures), and longitudinal regression modeling to assess the relationships between training variables, ECG classifications, and cardiovascular markers. Oracle Crystal Ball, a novel tool in this context, was employed for time-series forecasting, risk prediction, and Monte Carlo simulations, providing insights into future cardiac risk trends and the cost-effectiveness of PPE schedules.

## 3. Results

### 3.1. Descriptive Statistics

The mean training age was 8.55 years. The athletes trained approximately 9.82 times per week, with an average session duration of 3.01 h, leading to a mean weekly training volume of 29.43 h. In terms of biochemical markers, creatine kinase levels averaged 207.40 U/L, with a maximum of 312 U/L, reflecting training-induced muscle stress. Urea concentrations varied significantly, with a mean of 98 mg/dL and a maximum of 856.91 mg/dL, indicating potential variations in protein metabolism and hydration status. ALT levels remained within normal physiological limits, while serum magnesium and calcium levels were stable, with mean values of 2.00 mg/dL and 9.85 mg/dL, respectively.

The cohort displayed a lean body composition, with an average height of 1.51 m, a mean weight of 42.48 kg, and a BMI of 18.48. Cardiovascular assessments revealed an average systolic blood pressure of 103.47 mmHg and a diastolic blood pressure of 59.78 mmHg at rest. The mean resting heart rate was 66.72 bpm, and the aerobic capacity, as determined by VO_2_ max, averaged 52.01 mL/min/kg. Heart rate recovery post-exercise was recorded at 74.5 bpm, reflecting cardiovascular efficiency. A detailed overview of these variables is provided in Table 1.

Qualitative health-screening observations revealed that the majority of athletes were in the preparatory training period and exhibited normal heart rhythms with no murmurs. More than 90% of participants demonstrated normal ECG findings, and no athletes had a documented family history of cardiovascular disease. Among those with a prior SARS-CoV-2 infection, supplementary investigations, including echocardiography and cardiopulmonary exercise testing (CPET), revealed no residual cardiac abnormalities. Age-related variations in body composition were noted, with underweight status predominantly observed in younger athletes, whereas overweight classifications were exclusive to those over 16 years. These findings align with expected physiological adaptations influenced by training intensity and nutritional factors.

### 3.2. Statistical Analysis of Cardiovascular and Training Variables

The analysis revealed several relationships between the training variables and cardiovascular parameters. Training age and resting heart rate demonstrated a weak positive correlation (r = 0.111, *p* = 0.731), indicating no significant relationship and suggesting that factors beyond training age may influence resting heart rate. A negative, non-significant correlation (r = −0.416, *p* = 0.179) was observed between resting heart rate and training volume, suggesting a trend where athletes with higher training volumes tended to have lower resting heart rates. A significant positive correlation (r = 0.591, *p* = 0.043) was identified between resting heart rate and recovery heart rate, indicating that athletes with lower resting heart rates exhibited faster recovery times, which is a marker of superior cardiovascular efficiency.

Training age and VO_2_ max showed a negligible positive correlation (r = 0.030, *p* = 0.926), suggesting that VO_2_ max may be more influenced by current training intensity or genetic predisposition rather than cumulative training history. Creatine kinase levels varied widely (127–312 U/L), reflecting differential muscular stress and recovery patterns. However, non-significant correlations between creatine kinase levels and training variables suggested that creatine kinase is more indicative of acute muscular stress rather than cumulative training load.

The frequency analysis categorized athletes into two age groups, those below 16 years and those above 16 years, to identify differences in the prevalence of ECG parameters. This stratification provided insight into the developmental impact on the cardiac findings (Table 2).

Chi-square tests indicated several significant associations. BMI and age showed a highly significant relationship (*p* < 0.001), where underweight classifications were predominant in younger athletes. Overweight classifications were exclusive to athletes above 16 years. Bradycardia was significantly more prevalent in older athletes (*p* < 0.001), reinforcing the hypothesis of training-induced cardiac adaptations. Junctional escape rhythm was more frequently observed in older athletes (*p* < 0.001), suggesting that prolonged training influences conduction pathways. Premature atrial contractions (PAC) were significantly associated with training age (*p* = 0.045), indicating that prolonged training exposure may contribute to PAC occurrences. Incomplete right bundle branch block (IRBBB) demonstrated borderline significance (*p* = 0.051), suggesting a potential age-related trend that warrants further investigation.

**Figure 1:**
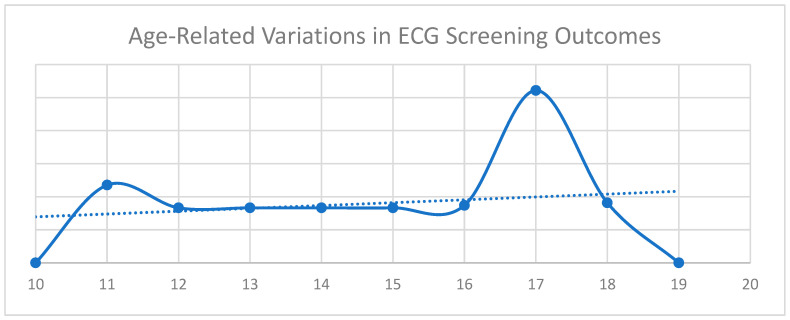
Age-related variations in ECG screening outcomes.

Figure 1 illustrates the relationship between age and ECG screening results based on international classification criteria [4]. ECG outcomes are categorized as 0—Normal, 1—Abnormal, and 2—Borderline. The *x*-axis represents age, while the *y*-axis denotes the corresponding ECG classifications. The continuous trendline highlights fluctuations in the ECG findings across age groups. Two distinct peaks are observed. The first corresponds to a higher occurrence of incomplete right bundle branch block (IRBBB), while the second is associated with an increased prevalence of T-wave inversions (TWI) in leads V1–V3.

The chi-square analysis examined the association between cardiovascular variables and age groups in athletes. A borderline-significant relationship was observed between IRBBB and age (*p* = 0.051, Pearson Chi-square), with the likelihood ratio test confirming significance (*p* = 0.047). Fisher’s exact test approached significance (*p* = 0.063, 2-tailed), indicating a potential trend in age-related cardiac remodeling due to cumulative training effects.

Bradycardia was significantly associated with age (*p* < 0.001, Pearson Chi-square), with a higher prevalence in older athletes (70%) compared to younger ones (30%). This result was supported by the likelihood ratio test (*p* < 0.001) and Fisher’s exact test (*p* = 0.001). These findings suggest training-induced adaptations, such as enhanced parasympathetic tone and increased stroke volume, in older athletes.

Respiratory sinus arrhythmia was more common in younger athletes (76.5% vs. 62.4%), though the association was not statistically significant (*p* = 0.073). Supporting tests, including the likelihood ratio (*p* = 0.067) and Fisher’s exact test (*p* = 0.081), reinforced this trend, suggesting potential age-related reductions in vagal tone.

A strong association was found between junctional escape rhythm and age (*p* < 0.001, Pearson Chi-square), with older athletes (69.6%) exhibiting a significantly higher prevalence compared to younger ones (30.4%). This result was confirmed by the likelihood ratio test (*p* < 0.001) and Fisher’s exact test (*p* < 0.001). The findings highlight potential age-dependent changes in cardiac conduction pathways influenced by prolonged training exposure.

EKG screening outcomes did not show significant associations with age (*p* = 0.281, Pearson Chi-square). Although older athletes had a higher prevalence of borderline and abnormal findings, the lack of statistical significance suggests that age alone may not be a determining factor in ECG screening results.

Premature atrial contractions (PAC) were significantly more frequent in the older age group (*p* = 0.045), possibly reflecting cumulative training effects on atrial electrical conduction. IRBBB demonstrated a borderline association with age (*p* = 0.051), implying a potential increase in prevalence due to training-induced cardiac remodeling. The occurrence of first-degree A-V block did not differ significantly across age groups (*p* = 0.384), but long-term monitoring remains crucial when evaluating cumulative training exposure effects.

**Figure 2:**
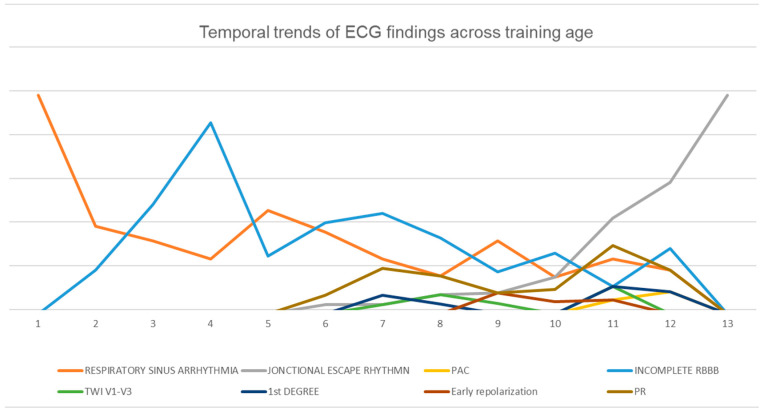
Temporal trends of ECG findings across training age.

Examining the relationship between training age and electrocardiographic (ECG) findings provides insight into cardiac adaptations in athletes. In Figure 2, the *x*-axis represents training age, while the *y*-axis denotes the prevalence of specific ECG patterns. Key parameters include respiratory sinus arrhythmia, junctional escape rhythm, premature atrial contractions (PAC), incomplete right bundle branch block (RBBB), T-wave inversion (TWI) in leads V1–V3, first-degree atrioventricular block, early repolarization, and PR interval variations.

Distinct age-related trends emerge from the data. Respiratory sinus arrhythmia declines progressively as the training age increases, while junctional escape rhythm becomes more prevalent in later training years. Incomplete RBBB reaches its highest occurrence during mid-training years before gradually decreasing. These findings underscore the dynamic nature of cardiac adaptations in athletes and emphasize the necessity of age-specific criteria when interpreting ECG results to differentiate between physiological adaptations and potential pathological concerns.

**Figure 3:**
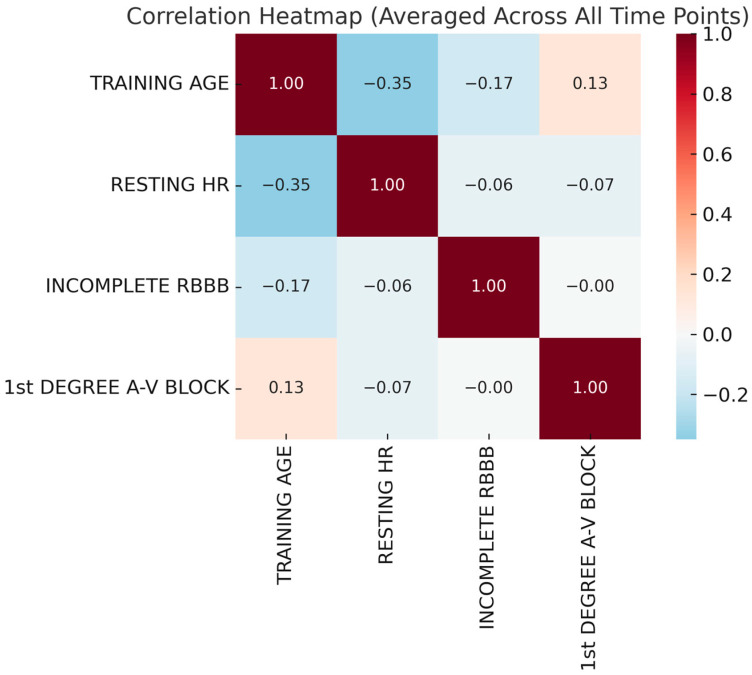
Correlation heatmap averaged across all time points.

The first heatmap (Figure 3) illustrates the correlations among training age, resting heart rate (HR), incomplete right bundle branch block (IRBBB), and first-degree atrioventricular (A-V) block across all time points. Correlation coefficients range from –1 to 1, with positive values indicating direct relationships and negative values suggesting inverse associations. A negative correlation between training age and resting HR suggests that increased training experience is associated with a lower resting HR, reflecting enhanced cardiovascular efficiency and autonomic adaptations. A weak positive correlation between training age and first-degree A-V block indicates that prolonged training may influence conduction patterns, potentially as part of athletic heart syndrome. The negative correlation between resting HR and first-degree A-V block supports the idea that athletes with a lower resting HR are more likely to exhibit conduction delays due to increased vagal tone. IRBBB showed minimal correlation with other variables, suggesting that it may be an incidental finding unrelated to training-related cardiac adaptations.

**Figure 4:**
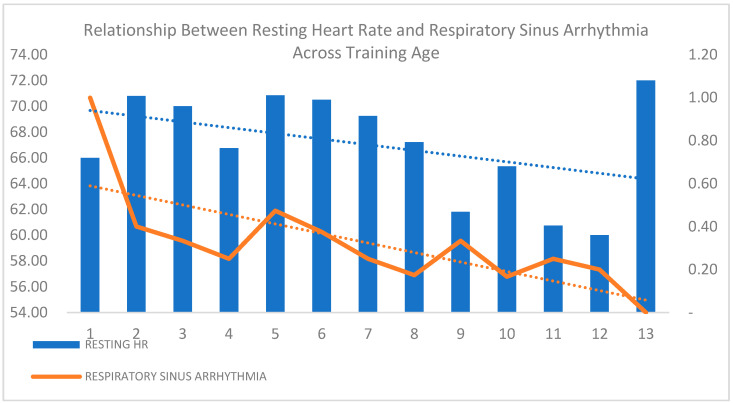
Relationship between resting heart rate and respiratory sinus arrhythmia across training age.

Respiratory sinus arrhythmia (RSA) was more prevalent in younger athletes, though the difference was not statistically significant (*p* = 0.073), suggesting a potential trend in autonomic cardiac modulation with age. Figure 4 illustrates the declining trend of resting heart rate (HR) and RSA as the training age increases, reflecting enhanced cardiovascular efficiency and autonomic adaptation in long-term athletes. The sharp decline in RSA highlights progressive parasympathetic modulation, which is consistent with the physiological adaptations seen in trained individuals. These findings suggest that prolonged training influences autonomic regulation, though further investigation is needed to confirm the clinical significance of these trends.

The logistic regression analysis identified significant predictors for various cardiovascular findings in athletes, including murmurs, arrhythmias, respiratory sinus arrhythmia, and incomplete right bundle branch block (IRBBB). The models incorporated training parameters (frequency, session duration, and weekly volume), biochemical markers (creatinine kinase, ALT, magnesium, calcium, total protein, urea, and glucose), and anthropometric measures (height, weight, and BMI). A stepwise entry method was applied, with predictors included at *p* < 0.05 and removed at *p* < 0.10.

The arrhythmia model was statistically significant (*p* = 0.002), explaining 60.3% of the variance (Nagelkerke R^2^ = 0.603) and achieving an overall classification accuracy of 96.9%. Elevated creatinine kinase levels (*p* = 0.007, OR = 1.020) were a significant predictor, suggesting a relationship between muscle metabolism and cardiac rhythm. Additionally, serum calcium (*p* = 0.040, OR = 0.111) and ALT levels (*p* = 0.014, OR = 0.667) were significantly associated, indicating metabolic and hepatic influences on conduction abnormalities.

The IRBBB prediction model was also statistically significant (*p* = 0.007) and explained 27.9% of the variance (Nagelkerke R^2^ = 0.279), with a classification accuracy of 74.8%. Weekly training volume (*p* = 0.022, OR = 20.250) emerged as a strong predictor, suggesting that higher training loads may contribute to IRBBB occurrence. Anthropometric factors also played a role, with BMI (*p* = 0.029, OR = 7.94 × 10^66^), height (*p* = 0.066, OR = 0.940), and weight (*p* = 0.023, OR = 0.000) significantly associated with conduction abnormalities.

A separate logistic regression model examining the relationship between age and resting heart rate (HR) demonstrated a moderate correlation (R = 0.33, R^2^ = 0.11), indicating that age accounts for 11% of HR variance. ANOVA results (F = 21.14, *p* < 0.001) confirmed a highly significant relationship. The negative regression coefficient (B = −0.05, *p* < 0.001) suggests that, with each additional year of age, the resting HR decreases by 0.05 bpm, supporting the concept of age-related cardiac adaptations. This decline likely reflects enhanced parasympathetic tone and increased stroke volume due to prolonged exposure to training, leading to improved cardiovascular efficiency.

The longitudinal analysis of ECG parameters in elite female gymnasts used a mixed-model ANOVA and a repeated-measures ANOVA to assess within-subject and between-subject variations over time. The mixed-model ANOVA captured individual variability, while the repeated-measures ANOVA analyzed temporal trends while correcting for sphericity violations.

The results from the repeated-measures ANOVA confirmed significant differences in the ECG parameters over time (*p* = 0.007). Mauchly’s test for sphericity (W = 0.000) indicated violations, requiring Greenhouse–Geisser corrections, with multivariate tests showing a strong time effect (*p* < 0.001).

Resting heart rate declined significantly over the 15 sessions (*p* = 0.006), with a linear trend (*p* = 0.004), suggesting training-induced cardiovascular adaptations. Notably, higher-order contrasts (*p* = 0.005) indicated fluctuations potentially linked to training intensity variations.

Training age had a highly significant effect (*p* < 0.001), reinforcing the progressive cardiovascular adaptations associated with cumulative training exposure.

IRBBB showed a significant time effect (*p* = 0.037), with non-linear variations (*p* = 0.022) possibly reflecting training-induced physiological stressors. Junctional escape rhythm followed a significant linear trend (*p* = 0.030), indicating gradual conduction system adaptations due to training loads. Respiratory sinus arrhythmia did not exhibit an overall time effect, but a significant 11th-order contrast (*p* = 0.008) suggested fluctuations influenced by training intensity peaks, rest periods, or psychological stressors. These findings highlight training-induced cardiac adaptations and the importance of longitudinal ECG monitoring in athletes. The correlation analysis from the Astrand test in elite female gymnasts examined key cardiovascular fitness parameters, including resting heart rate (HR), HR at minute 6, recovery HR, VO_2_ max, and aerobic fitness scores to assess the relationships between exercise-induced responses and aerobic capacity.

A strong negative correlation was found between HR at minute 6 and VO_2_ max (r = −0.65 to –0.78, *p* < 0.05), indicating that athletes with a higher aerobic capacity exhibited lower HR during submaximal exercise, reflecting greater cardiovascular efficiency. Similarly, resting HR also showed an inverse correlation with VO_2_ max, reinforcing the link between a lower resting HR and an improved aerobic fitness.

Recovery HR post-exercise demonstrated significant inverse correlations with VO_2_ max (r = –0.60 to –0.82, *p* < 0.05), suggesting that a faster recovery HR is associated with greater aerobic capacity. This highlights recovery HR as a sensitive marker of cardiovascular adaptation and a potential indicator of overtraining or maladaptation when deviations occur.

Training age showed moderate positive correlations with aerobic fitness scores, indicating that prolonged training leads to measurable cardiovascular adaptations. However, correlation strength varied among individuals, likely due to differences in training loads, genetic predispositions, recovery capacity, and adaptation rates.

**Figure 5:**
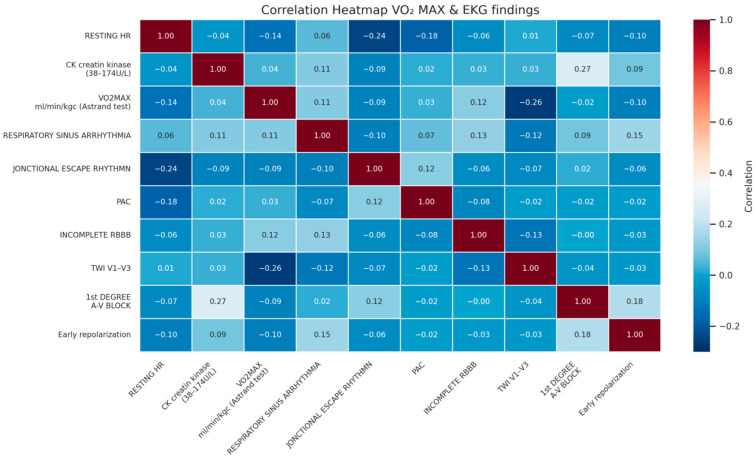
Correlation heatmap of Vo2 max and ECG findings.

Higher aerobic capacity appears to be linked to distinct cardiac adaptations, as reflected in the correlation heatmap. A negative correlation (–0.14) between VO_2_ max and resting heart rate (HR) suggests that athletes with a greater aerobic fitness tend to have a lower resting HR, an indicator of enhanced cardiovascular efficiency. Similarly, T-wave inversions (TWI) in leads V1–V3 showed a moderate inverse correlation (–0.26) with VO_2_ max, implying that these ECG patterns may be less common in athletes with superior aerobic conditioning (Figure 5). A moderate negative correlation (–0.24) between resting HR and junctional escape rhythm indicates that lower HR values may be associated with an increased prevalence of this rhythm, likely due to autonomic adaptations from training. Additionally, creatine kinase (CK) levels correlated mildly (0.27) with first-degree A-V block, suggesting a potential association between higher muscle stress and cardiac conduction variations.

**Figure 6:**
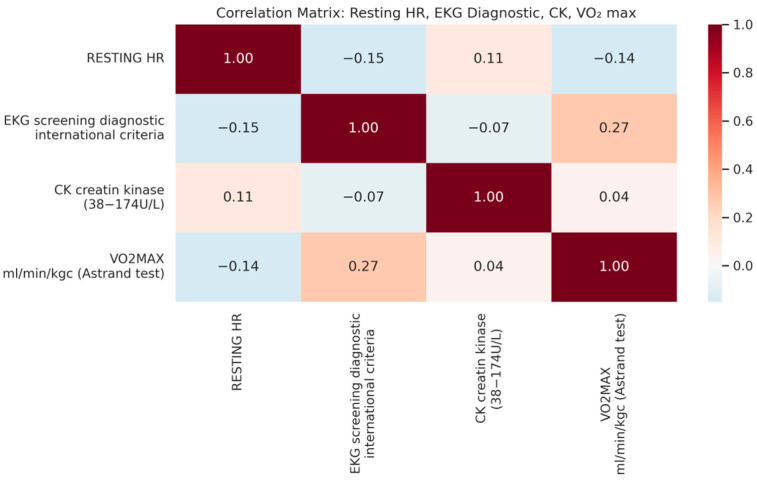
Correlation matrix: resting HR, ECG diagnostic, CK, and Vo2 max.

Variations in resting heart rate (HR), ECG screening diagnostics, creatine kinase (CK) levels, and VO_2_ max reveal important trends in athletic cardiovascular adaptation. A slight positive correlation (0.27) between VO_2_ max and ECG diagnostics suggests that certain ECG patterns may be linked to aerobic capacity, possibly reflecting training-induced cardiac remodeling (Figure 6). A weak negative correlation (–0.14) between resting HR and VO_2_ max aligns with expectations, as athletes with better cardiovascular fitness typically exhibit a lower resting HR. In contrast, the CK levels showed minimal correlation with ECG patterns and VO_2_ max, indicating that muscle stress markers may not directly influence cardiac adaptations in this cohort. These findings reinforce the complex interplay between training, cardiovascular efficiency, and electrical activity of the heart.

The correlation analysis between training volume, resting heart rate (HR), and HR after standing revealed significant associations. A higher training volume correlated negatively with resting HR (r = –0.685 to −0.722, *p* < 0.005), suggesting enhanced cardiovascular efficiency. Similarly, training volume negatively correlated with HR after standing (r = –0.602 to –0.537, *p* < 0.05), indicating better autonomic regulation in well-trained athletes. A strong positive correlation between resting HR and HR after standing (r = 0.794 to 0.933, *p* < 0.001) suggests that athletes with higher resting HR exhibit greater HR increases upon standing, potentially reflecting reduced autonomic adaptability. These findings emphasize the role of training load in cardiovascular regulation, though individual variability was observed.

**Figure 7:**
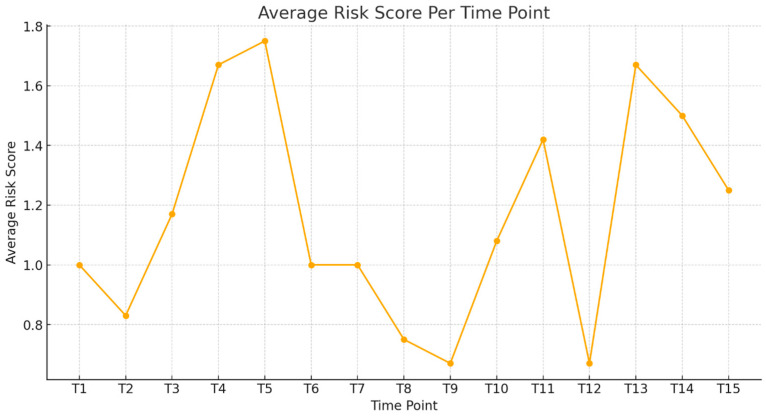
Average risk score per time point.

Figure 7 illustrates the evolution of the ECG findings across multiple assessment periods (T1–T15) in the cohort, providing insight into training-related cardiac adaptations over time. Elevated scores at T4–T5 suggest periods of increased training intensity or physiological stress, while the decline after T6 likely reflects cardiovascular adaptations and improved autonomic regulation. By T12–T15, consistently lower scores may indicate stabilized cardiac responses or reduced physiological strain. This score serves as a longitudinal monitoring tool, tracking training-related ECG variations. Threshold values (e.g., score > 2) may signal the need for further cardiological assessment, while predictive modeling can help estimate athlete readiness and long-term cardiac trends, ensuring optimal cardiovascular management.

Scoring System Explanation

The risk score was assigned by allocating one point for each ECG anomaly, one point for borderline ECG classifications, and two points for abnormal ECG classifications per athlete at each time point.

### 3.3. Predictive Modeling and Monte Carlo Simulations

Forecasting Resting Heart Rate Using Double Exponential Smoothing

A time-series forecast of resting heart rate (HR) across 13 observed periods (T1–T13) was conducted using Oracle Crystal Ball, extending predictions for an additional 10 periods (T14–T23) through double exponential smoothing. This method was selected based on performance metrics, with alpha (smoothing level) = 0.6852 and beta (trend smoothing) = 0.1378 optimizing trend estimation.

The historical HR data exhibited a mean of 66.73 bpm, ranging from 60.83 bpm to 73.33 bpm, with a standard deviation of 3.95 bpm. The model demonstrated high forecast accuracy, as indicated by a root mean square error (RMSE) of 2.07, a Theil’s U statistic of 0.9461, and a Durbin–Watson statistic of 1.96, suggesting minimal autocorrelation.

The forecast projected a gradual decline in mean HR, from 61.16 bpm at T14 to 54.95 bpm by T23, reinforcing the trend of training-induced bradycardia. The 95% confidence intervals expanded over time, with predicted values ranging from 39.18 bpm (lower bound at T23) to 70.72 bpm (upper bound at T23), reflecting increasing uncertainty with extended projections.

**Figure 8:**
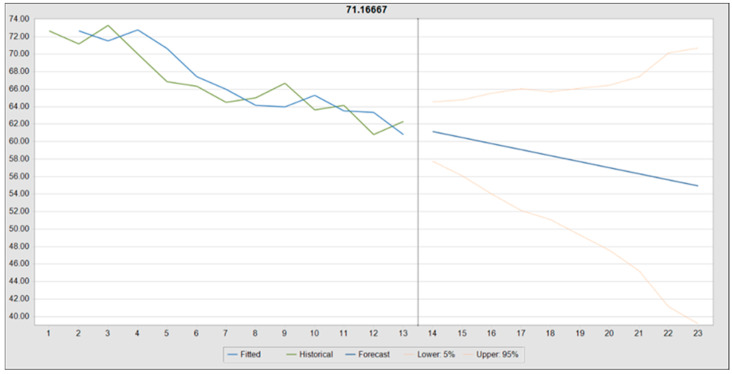
Forecasted resting heart rate trend using double exponential smoothing.

Alternative models, including damped trend non-seasonal and single exponential smoothing, produced comparable RMSE values (2.07 and 2.16, respectively), though double exponential smoothing exhibited superior trend adaptability. Figure 8 provides a visual representation of the projected HR trajectory, including historical data, the predicted trend, and confidence intervals, emphasizing the model’s stability and widening prediction range over time.

A time-series forecast of a single athlete’s ECG classifications was conducted to assess individual variability and detect early trends in abnormal readings. The ARIMA (0, 0, 1) model was chosen for its ability to capture short-term fluctuations effectively, predicting a 50% likelihood of recurrent abnormal ECG classifications over the next five periods (T16–T20). The model demonstrated moderate predictive accuracy, with an RMSE of 0.33 and Theil’s U value below one, confirming reliable forecasting performance. Minimal autocorrelation, as indicated by a Durbin–Watson statistic near two, suggests the absence of systematic bias in the residual errors. Additionally, the narrow confidence interval enhances confidence in the projected trend.

**Figure 9:**
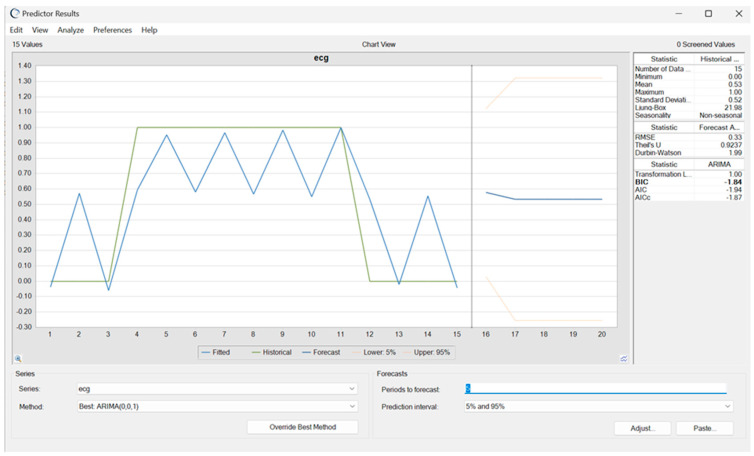
Forecasted ECG classifications for a single athlete using ARIMA modeling.

The model’s predictions align with the historical fluctuations observed between T4 and T11, where recurring abnormal ECG patterns may have been influenced by training loads, cardiac remodeling, or transient physiological adaptations. While the mid-range probability (~0.5) suggests a transient rather than a persistent pathological risk, continuous monitoring remains essential for refining individualized cardiac risk assessments (Figure 9).

A Monte Carlo simulation was conducted to compare the cost-effectiveness of pre-participation evaluations (PPEs) every 6 months versus every 12 months among youth athletes. The 6-month schedule involved 168 total assessments (12 athletes assessed 14 times), whereas the 12-month schedule included 84 total assessments (12 athletes assessed 7 times).

The 6-month PPE schedule incurred a total cost of EUR 25,000, with a cost per necessary test of EUR 7840 and an unnecessary testing rate of 70%. In contrast, the 12-month schedule was more cost-efficient, with a total cost of EUR 6700, a cost per necessary test of EUR 5880, and a higher unnecessary testing rate of 83.33%. Extra testing costs were highly variable under the 6-month scenario (mean EUR 8.89, skewness = 2.74), whereas the costs in the 12-month scenario were more stable but higher per test (mean EUR 5849.81, range: EUR 4500.09–EUR 7606.09).

Break-Even Analysis

A break-even analysis was performed to determine the minimum number of detected cardiac abnormalities needed to justify the costs of each PPE schedule. The 6-month scenario incurred a total cost of EUR 25,000 (EUR 23,520 in PPE costs and EUR 1480 in extra testing costs), while the 12-month scenario totaled EUR 6,700 (EUR 5880 in PPE costs and EUR 820 in extra testing costs). This resulted in a cost per problem detected of EUR 7840 for the 6-month schedule and EUR 5880 for the 12-month schedule, indicating that more frequent screening does not substantially improve cost efficiency.

These findings suggest that a 12-month PPE schedule is the more cost-effective approach, despite a higher proportion of unnecessary tests. Increasing screening frequency to every 6 months does not significantly enhance the identification of clinically relevant cases and, instead, leads to higher overall costs.

## 4. Discussion

This study provides critical longitudinal insights into the cardiovascular and metabolic adaptations of elite female gymnasts, systematically addressing the study’s three objectives and the four original hypotheses. By leveraging diverse statistical approaches, including Pearson correlation, Chi-square tests, logistic regression, ANOVA, and predictive modeling through Oracle Crystal Ball, this research illuminates the nuanced effects of long-term training on cardiac function, the stability of ECG patterns, and the economic considerations surrounding PPE screening intervals.

The findings confirm significant cardiovascular adaptations in elite gymnasts over time, aligning with the first objective. Resting heart rate demonstrated a consistent decline across multiple PPE cycles, reflecting enhanced parasympathetic regulation and improved cardiac efficiency—characteristics of well-conditioned athletes. These results reinforce the prior literature suggesting that long-term, high-intensity training reduces baseline heart rate and improves recovery, key markers of autonomic fitness [4,7,8].

Age-related changes in ECG findings further validated the hypothesis that sustained training leads to physiologic cardiac remodeling [9]. Older athletes displayed a higher prevalence of bradycardia, indicating increased parasympathetic tone and augmented stroke volume. Junctional escape rhythms were more common in these athletes, suggesting cumulative training-induced influences on conduction pathways. Similarly, T-wave inversions in leads V1–V3, often described as juvenile patterns, appeared more frequently after the age of 16, highlighting the need for age-specific ECG interpretation to avoid the misclassification of normal training-induced changes as pathological [10].

While IRBBB was observed at borderline significance, its presence—typically benign in trained individuals—underscores the importance of ongoing longitudinal monitoring. IRBBB patterns might be influenced by cumulative training stress and should be tracked over time to distinguish stable adaptations from potential early signs of conduction abnormalities. The data also revealed a general stability in other ECG parameters, such as PR interval and early repolarization, emphasizing that many features remain consistent despite intensive training. This aligns with prior findings that early repolarization (ER) is commonly observed in athletes and is often considered a benign variant, particularly in highly trained individuals with increased vagal tone [11].

Further, by correlating the ECG findings with training volume, frequency, and session duration, the results indicated that cumulative training exposure promotes physiological cardiac remodeling without evidence of progressive pathology. Although the biochemical markers (e.g., CK and ALT) did not exhibit significant correlations with the ECG changes, heatmap analyses provided additional insight into the relationships among training variables, heart rate metrics, and ECG patterns.

The heatmap analysis provided additional perspective on the interrelationship between training adaptations and ECG changes, reinforcing the broader findings of this study. The observed negative correlation between VO_2_ max and resting heart rate aligns with prior research demonstrating a significant relationship between VO_2_ max and left ventricular mass in endurance-trained athletes, reinforcing the impact of aerobic conditioning on structural cardiac adaptations [12]. Additionally, the inverse relationship between VO_2_ max and T-wave inversions (TWI) in leads V1–V3 suggests that, as aerobic fitness improves, juvenile patterns may become less frequent, emphasizing the need for age-specific ECG interpretation in elite athletes. The negative correlation between resting HR and junctional escape rhythm highlights increased vagal tone as a normal training effect rather than an indication of pathology, while the mild association between creatine kinase and first-degree A-V block suggests a potential link between muscle stress and conduction variations. These findings further underscore the complexity of ECG interpretation in trained athletes, where individual variability and training history must be considered to avoid the misclassification of physiological adaptations as pathological findings.

Overall, these results support the hypothesis that sustained, intensive training induces stable yet significant cardiovascular adaptations, reflecting the body’s ability to maintain cardiac resilience and efficiency over time. Similar findings have been reported in female Olympic athletes, where training-induced electrical and structural remodeling was observed but to a lesser degree compared to male athletes [13].These findings align with the concept of the athlete’s heart, where cumulative training results in structural and functional changes that are non-pathological and indicative of superior cardiovascular performance.

The use of Oracle Crystal Ball introduced a novel approach to predicting future ECG trends, fulfilling the second study objective. By identifying athletes at higher risk for cardiac abnormalities, these models enable a more targeted follow-up, potentially reducing the need for unnecessary tests and interventions. The ability to anticipate declines in resting heart rate and pinpoint outlier values demonstrates the potential for personalized risk assessments. These findings confirm the utility of combining historical data with advanced forecasting methods to inform clinical decisions and enhance early intervention strategies.

The comparison of 6-month versus 12-month PPE intervals revealed important economic implications, addressing the third study objective. The Monte Carlo simulations demonstrated that a 12-month schedule is more cost-effective, achieving a break-even cost threshold after detecting just one cardiac event compared to three events in the 6-month scenario. While the 12-month interval had a higher proportion of unnecessary tests, the overall cost savings and comparable diagnostic sensitivity suggest that it remains a practical option for elite athletes.

However, these findings do not imply that all PPE should be performed annually, particularly for high-risk populations or athletes presenting new symptoms. Rather, they highlight the value of refining screening strategies by integrating predictive models and larger datasets. Future research should explore how decision-tree models and expanded datasets could further optimize PPE frequency, balancing financial considerations with the paramount goal of safeguarding athlete health.

Building upon the advancements in AI-ECG analysis and refined diagnostic approaches for the athlete’s heart, as highlighted by the use of Z-scores for precise screening, the integration of predictive analytics and cost-effectiveness analyses represents a crucial next step in sports cardiology [14,15,16,17] By leveraging Oracle Crystal Ball and focusing on meaningful physiological markers, this study provides a framework for improving athlete safety, reducing unnecessary testing, and advancing the precision of cardiac monitoring protocols in elite sports. Future efforts should aim to validate these models in larger cohorts and refine screening intervals based on individualized risk profiles, ultimately advancing both the scientific understanding and clinical management of athletic cardiac health.

The findings of this study collectively underscore the importance of continuous cardiac monitoring and personalized risk assessment in elite athletes. The stability of most ECG parameters over time supports the reliability of initial screenings in predicting long-term cardiac profiles. Simultaneously, the presence of occasional abnormal findings in some athletes reinforces the need for ongoing vigilance to identify the conditions requiring clinical intervention.

This study’s relatively small cohort, comprising elite athletes from a single national center, limits generalizability to other populations. The retrospective data collection may have introduced variability in documentation and assessment conditions, potentially influencing observed trends. Additionally, the absence of a pathological comparison group, such as athletes with known cardiac conditions or recreational athletes, makes it difficult to definitively distinguish physiological adaptations from potential pathology. Lastly, the predictive models, while novel, were based on a homogenous dataset. Future research should expand the sample sizes, include a control group, and validate their findings with larger and more diverse cohorts to enhance generalizability and predictive accuracy.

## 5. Conclusions

This study revealed training-induced cardiovascular adaptations in elite female gymnasts, with significant findings including the progressive reduction in resting heart rate, increased prevalence of junctional escape rhythms, and T-wave inversions post-age 16. These changes reflect the physiological cardiac remodeling associated with cumulative training exposure and underscore the importance of age-specific interpretation when assessing ECG findings in young athletes. Predictive modeling using Oracle Crystal Ball demonstrated the feasibility of anticipating ECG trends and identifying higher-risk athletes. Although training load was not a significant predictor, the models offer a foundation for refining the risk stratification when applied to larger datasets with additional variables. Monte Carlo simulations and a break-even analysis confirmed that less-frequent screening remains clinically effective. However, screening strategies should balance economic efficiency with the life-saving potential of early detection. A future cost–utility analysis (CUA) would help ensure that decisions remain both evidence-based and patient-centered. In conclusion, this study validates the stability of ECG profiles over time, demonstrates the potential of predictive analytics to enhance athlete safety, and presents a framework for cost-effective cardiac screening that does not compromise diagnostic accuracy. By advancing both scientific understanding and clinical practice, this research paves the way for refined screening strategies, better-informed risk assessments, and ultimately, improved cardiac health management in elite athletes.

## Figures and Tables

**Table 1:** Descriptive statistics of cardiovascular, training-related, biochemical, and anthropometric parameters in elite female gymnastics athletes.

Variable	Minimum	Maximum	Mean	Std. Deviation
Training Age	5.73	10.27	8.5508	1.51395
Training Sessions per Week	8.07	10.53	9.8246	0.64087
Training Hours per Week	2.83	3.4	3.0119	0.16376
Training Volume	24.73	34.27	29.4327	2.47527
CK	127	312.1	207.4046	54.68016
ALT	17.22	26.91	21.7608	3.30324
Mg	1.84	2.15	2.0043	0.10573
Ca	9.58	10.13	9.853	0.16894
Total Protein	68.64	74.22	71.3766	1.60315
Urea	25.5	856.91	98.118	238.98411
Glucose	85.64	96.33	91.234	3.44214
Height	1.44	1.58	1.5064	0.04317
Weight	36.71	48.36	42.4813	3.64275
BMI	16.9	21.94	18.4829	1.28455
Body Fat	10.22	19.41	12.0874	2.43639
FFM%	82.13	89.78	88.0412	2.01967
SBPmmHg REST	98.57	107.67	103.4722	2.44312
DBPmmHg REST	54.29	63.67	59.7837	2.37813
HR bpm REST	60.4	73.6	66.7246	3.74571
SBPmmHg	100	106	104.0317	1.79075
DBPmmHg	60	66	62.3313	1.56441
HR Min 6 Astrand Test	137.14	147.6	141.6431	3.20956
HR Recovery	62	84	74.4685	5.96877
VO_2_ Max	43.38	56.18	52.0109	3.10713
Aerobic Fitness	0.2	1	0.8312	0.22715
RUFFIER index	3.85	7.27	5.4086	0.99107

**Table 2:** Distribution of ECG parameters across age groups.

Measure		Below 16		Above 16		Total		χ^2^ Test
Training Period	Preparatory	91	64.08%	51	35.92%	142	80.68%	0.169
	Precompetitive	26	76.47%	8	23.53%	34	19.32%	
BMI Status	Underweight	82	98.80%	1	1.20%	83	47.16%	*p* < 0.001
	Normal	35	39.33%	54	60.67%	89	50.57%	
	Overweight	0	0.00%	4	100.00%	4	2.27%	
RATE	Bradycardia	6	30.00%	14	70.00%	20	11.36%	*p* < 0.001
	Normal	111	71.15%	45	28.85%	156	88.64%	
Respiratory Sinus Arrhythmia	Absent	78	62.40%	47	37.60%	125	71.02%	0.73
	Present	39	76.47%	12	23.53%	51	28.98%	
Junctional Escape Rhythm	Absent	110	71.90%	43	28.10%	153	86.93%	*p* < 0.001
	Present	7	30.43%	16	69.57%	23	13.07%	
PAC	Absent	117	67.24%	57	32.76%	174	98.86%	0.045
	Present	0	0.00%	2	100.00%	2	1.14%	
IRBBB	Absent	72	61.54%	45	38.46%	117	66.48%	0.051
	Present	45	76.27%	14	23.73%	59	33.52%	
TWI V1–V3	Absent	117	68.82%	53	31.18%	170	96.59%	*p* < 0.001
	Present	0	0.00%	6	100.00%	6	3.41%	
1st AV block	Absent	114	67.06%	56	32.94%	170	96.59%	0.384
	Present	3	50.00%	3	50.00%	6	3.41%	
EKG Screening	Normal	107	67.72%	51	32.28%	158	89.77%	0.281
	Borderline	0	0.00%	1	100.00%	1	0.57%	
	Abnormal	10	58.82%	7	41.18%	17	9.66%	
PR Interval	Short	10	90.91%	1	9.09%	11	6.25%	0.153
	Normal	104	65.41%	55	34.59%	159	90.34%	
	Long	3	50.00%	3	50.00%	6	3.41%	
Valvular Heart Disease	Absent	117	66.48%	59	33.52%	176	100.00%	

## Data Availability

The data that support the findings of this study are available from the corresponding author upon request.

## Abbreviations

The following abbreviations are used in this manuscript:

A.V. Block	Atrioventricular Block
ALT	Alanine Aminotransferase
ANOVA	Analysis of Variance
ARIMA	Autoregressive Integrated Moving Average
BMI	Body Mass Index
BP	Blood Pressure
CK	Creatine Kinase
CPET	Cardiopulmonary Exercise Testing
DBP	Diastolic Blood Pressure
ECG	Electrocardiogram
ER	Early Repolarization
FFM%	Fat-Free Mass Percentage
HR	Heart Rate
IRBBB	Incomplete Right Bundle Branch Block
PAC	Premature Atrial Contractions
PPE	Pre-Participation Evaluation
PR	PR Interval
RSA	Respiratory Sinus Arrhythmia
SBP	Systolic Blood Pressure
TWI	T-Wave Inversion
VO_2_ max	Maximal Oxygen Uptake
